# Patterns of *Plasmodium vivax *and *Plasmodium falciparum *malaria underscore importance of data collection from private health care facilities in India

**DOI:** 10.1186/1475-2875-8-227

**Published:** 2009-10-12

**Authors:** Sangeeta Gupta, James T Gunter, Robert J Novak, James L Regens

**Affiliations:** 1 College of Public Health, University of Oklahoma Health Sciences Center, Oklahoma City, Oklahoma, USA; 2 College of Medicine, University of Alabama, Birmingham, Alabama, USA

## Abstract

**Background:**

This study describes patterns of falciparum and vivax malaria in a private comprehensive-care, multi-specialty hospital in New Delhi from July 2006 to July 2008.

**Methods:**

Malarial morbidity by Plasmodium species (*Plasmodium falciparum, Plasmodium vivax*, or *Plasmodium sp*.) was confirmed using microscopy and antigen tests. The influence of seasonal factors and selected patient demographics on morbidity was evaluated. The proportions of malaria cases caused by *P. falciparum *at the private facility were compared to data from India's National Vector Borne Disease Control Programme (NVBDCP) during the same period for the Delhi region.

**Results:**

In New Delhi, *P. faciparum *was the dominant cause of cases requiring treatment in the private hospital during the period examined. The national data reported a smaller proportion of malaria cases caused by *P. falciparum *in the national capital region than was observed in a private facility within the region. *Plasmodium vivax *also caused a large proportion of the cases presenting clinically at the private hospital during the summer and monsoon seasons.

**Conclusion:**

The proportion of *P. falciparum *malaria cases tends to be greatest during the post-monsoon season while the proportion of *P. vivax *malaria cases tends to be greatest in the monsoon season. Private hospital data demonstrate an under-reporting of malaria case incidences in the data from India's national surveillance programme during the same period for the national capital region.

## Background

Although malaria is a serious public health problem in many developing countries, estimates of the number of malaria cases and deaths frequently have lacked sufficient accuracy for establishing reliable baselines against which to evaluate the success of control measures. Three factors affect whether routinely reported malaria data accurately reflect patterns in malaria in countries with surveillance programmes, even ones that have relatively good statistical services [1]. First, information in routine surveillance systems may be incomplete. This may result in overestimates of reporting completeness thereby underestimating malaria patterns. Second, patients may use private health care facilities for treatment, especially in the South-East Asia region. Many malaria cases treated at private facilities are not included in the official statistics. Third, in some developing countries, diagnosis is based predominantly on clinical presentation with only a small proportion of suspected malaria cases subjected to laboratory verification. Even if laboratory testing is used for diagnosis, the validity and reliability of the test may influence the diagnosis. For example, antigen tests in conjunction with microscopy provide a more accurate identification of the *Plasmodium *species than blood slides. Each of these factors may generate errors in reported cases and need to be taken into consideration when estimating patterns of malaria in a country.

In 2006, for example, India's National Vector Borne Disease Control Programme (NVBDCP) reported approximately two million malaria cases based on surveillance carried out by India's primary health care system [2]. However, a recent analysis indicates there is a 68% to 98% gap between India's reported malaria cases and the actual incidence of malaria [3]. The difference between the reported number of cases and the 'true' number of incidents may stem from the fact that the government health sector meets the needs of 20% of the population, leaving 80% of the population to seek health care in the private facilities (Kumar *et al *2007). This is consistent with a recent WHO report which indicates that the real burden of malaria in India has been underestimated primarily due to reporting factors [1]. This underscores the importance of including private sector data to generate estimates that are more accurate.

In order to help bridge this gap, this paper examines patterns of *Plasmodium vivax *and *Plasmodium falciparum *malaria based on data from a private comprehensive-care, multi-specialty hospital in Delhi for a 24-month period from July 2006 through July 2008. Malarial morbidity was analysed by Plasmodium species and confirmed using microscopy and antigen tests. Seasonal factors and selected patient demographics are considered. To place these results in context, the proportion of malaria cases caused by *P*. *falciparum *at the private facility are compared to the NVBDCP data.

## Methods

### Study site

The hospital chosen for this study was a private comprehensive-care, multi-specialty tertiary care hospital in New Delhi, the capital of India. It provides a complete range of the latest diagnostic, medical, and surgical facilities for the care of its patients. Private hospitals like the one which provided patient data for this study attract patrons from all over India as well as abroad, depending on the financial resources available to the patient or his/her family, the type of medical treatment sought, and the availability of comparable facilities. This makes delineating the overall catchment area for the population served by the facility problematic. However, because the focus is on malaria and the hospital that provided the data in the National Capital Region (NCR), it is reasonable to assume that the NCR is the primary catchment area. The NCR refers to the metropolitan area, which encompasses the entire National Capital Territory of Delhi (NCT), as well as the urban areas surrounding it in the neighbouring states of Haryana, Uttar Pradesh, and Rajasthan. With a total area of about 33,578 km^2 ^(12,965 mi^2^), it is one of world's largest urban aggregations. In 2001, the NCT population was approximately 9.81 million with a ratio of 925 women per 1,000 men.

Table 1 lists the malaria cases and available demographic, *Plasmodium sp*., and temporal information used for analysis. To protect patient confidentiality, patient records were not provided. Only summary demographic information for malaria patients was provided including gender and age group.

**Table 1:** Malaria cases by *Plasmodium *species from a private tertiary care hospital in New Delhi

**Species**	**Total**	**Gender**		**Age Group**		**Season**			
		**Male**	**Female**	**Pediatric**	**Adult**	**Summer**	**Monsoon**	**Post-Monsoon**	**Winter**
*Plasmodium falciparum*	56	43	13	7	49	3	26	22	5
*Plasmodium vivax*	26	20	6	3	23	4	19	3	0
Unknown *Plasmodium*	31	20	11	2	29	4	14	8	5
Total	113	83	30	12	101	11	59	33	10

### Seasons

Seasonal categories were used to describe patterns in the occurrence of malaria cases. The climate of Delhi is semi-arid with high variation between summer and winter temperatures. The year is characterized by four seasons: summer, monsoon, post-monsoon, and winter. Summer lasts from March to June. Temperatures average around 32-40°C (90-104°F) in most of the interior. The monsoon (rainy) season lasts from July to September. This season is dominated by the humid southwest monsoon, which slowly sweeps across the country beginning in late May or early June. Monsoon rains begin to recede from North India at the beginning of October. The post monsoon season lasts from October to December, marking the beginning of the post monsoon season. The year's coldest months are December and January, when temperatures average around 10-15°C (50-59°F), with winter occurring between January and March.

### Plasmodium species

Because the protozoan parasites of the genus *Plasmodium*, which cause malaria require warm, humid environments for replication in the insect vector, the four malaria-generating species are mostly limited to tropical and sub-tropical locations. All are transmitted to human hosts through blood-feeding by *Anopheles *mosquito vectors. *Plasmodium falciparum *is the most widespread in tropical and sub-tropical areas. It is considered the most dangerous of these parasites because the highest rates of complications and mortality are associated with *P. falciparum *infections and is primarily targeted by the NVBDCP. *Plasmodium vivax *occurs throughout India and is a major contributor to the burden of malaria in the country. *Plasmodium ovale *is most prevalent in the west coast region of Africa while *Plasmodium malariae *is found worldwide. *Plasmodium malariae *causes "benign malaria" and is considered not as dangerous as malaria caused by *P. falciparum *or *P. vivax*.

For this study, diagnosis of *Plasmodium *species was done using microscopy and antigen tests. The antigen tests to determine *Plasmodium *species are based on the detection of specific antigens derived from malaria parasites in lysed blood using immunochromatographic methods. Two types of antigen tests were used: histidine-rich protein II (HRP-II) was used for the detection of *P. falciparum *and parasite lactate dehydrogenase (pLDH) was used to detect the non-falciparum *Plasmodium *species. HRP- II is a water-soluble protein produced by trophozoites and young (but not mature) gametocytes of *P. falciparum*. Parasite lactate dehydrogenase is produced by asexual and sexual stages (gametocytes) of malaria parasites. This test can accurately distinguish *P. falciparum *from the non-falciparum species, but cannot differentiate between *P. vivax, P. ovale *and *P. malariae*. Hence, microscopy was done to corroborate with antigen tests in all cases. Positive species identification was done using microscopy. Cases where species could not be determined definitively were labeled as unspecified.

### Malaria complications

Malaria can cause various serious complications involving various systems of the body. For this study, patients were categorized in terms of whether they experienced malaria complications using modified WHO Criteria which define severe malaria as asexual forms of *P. falciparum *in a peripheral blood film plus one of the following signs [4]:

1. Cerebral malaria: Glasgow coma scale < 10/14;

2. Severe anaemia: haematocrit < 20% plus parasite count of > 100,000/mm^3 ^in peripheral blood smear;

3. Renal failure: urine output < 400 ml/day plus a serum creatinine of > 3 mg/dL;

4. Pulmonary oedema

5. Jaundice: Serum bilirubin >3 mg/dL plus either parasite count > 100,000/mm^3 ^in a peripheral blood film or renal failure-serum creatinine > 1.5 mg/dL;

6. Hypoglycaemia: whole blood glucose < 40 mg/dL;

7. Circulatory collapse: Systolic blood pressure < 70 mm of Hg with cold clammy skin or core - skin temperature difference > 10°C;

8. Hyperparasitaemia: > 10%;

9. Spontaneous bleeding/DIC;

10. Acidaemia/Acidosis pH < 7.25 or plasma bicarbonate 2 in 24 hours

Complicated cases were placed in two categories: very severe cases exhibiting cerebral malaria, and cases with any other complications.

### Data collection and statistical techniques

Data were collected for all cases admitted with a primary diagnosis of suspected of known malaria admitted for treatment during the 24-month period between July 2006 and July 2008. A clinical diagnosis of malaria was determined by either a health care professional who referred the patient to the hospital for treatment or by a attending hospital physician. This initial diagnosis was confirmed by the antigen tests and microscopy described previously. The data collected includes the patient's demographic profile (age, gender), *Plasmodium *species, admission date, and presence of complications. A retrospective analysis was done to study the seasonal patterns in malaria cases admitted to this hospital during the two-year study period using SAS statistical package version 9.1.3 [5].

Frequency analyses were used to describe the differences in case prevalence by *Plasmodium *species based on age groups, gender, and month of occurrence (e.g., temporal variation by season). Age was treated as both a continuous variable (actual age) and categorical variable (paediatric/adult) in the statistical analysis. In Indian hospitals, the paediatric group is defined as ≤ 12 years old so this cut-off point was used for categorization of children and adults in the patient population. The number of adult patients was much greater than the number of paediatric patients, and more males had malaria than females. Because the number of cases involving children and female patients was limited, caution is warranted in making any age and\or gender inferences.

A variation of Fisher's exact test suitable for tables larger than 2 x 2 developed by Freeman and Halton was used with contingency tables to test for independence between *Plasmodium *species and seasons [5]. The Freeman and Halton variant of Fisher's exact test employs a hyper-geometric distribution derived from all possible combinations using the row and column marginal totals to generate an empirical distribution of the data. That distribution is then used to determine the probability of cell outcomes, considering all possible cell values that can result in equivalent marginal totals. The test adjusts for the table cells with a small number of observations to accurately reflect the underlying χ^2 ^distribution. The Freeman and Halton test calculates all tables with a probability less than or equal to the probability of the observed table. The null hypothesis is no association exists between season and *Plasmodium *species. A small p-value indicates the null hypothesis should be rejected. The Freeman and Halton test identifies only general, and not linear, associations, and does not provide right or left-sided p-values.

The Cochran-Armitage Test for trend was used to evaluate whether there was a statistically significant trend between *Plasmodium *species and the season of occurrence [6,7]. The test for trend determined if the proportion of cases in each of the seasons (ordered from spring through winter) increased or decreased in a linear fashion for the different *Plasmodium *species (*P. falciparum*, *P. vivax*, or unidentified species) [5,7].

## Results and discussion

**Figure 1:**
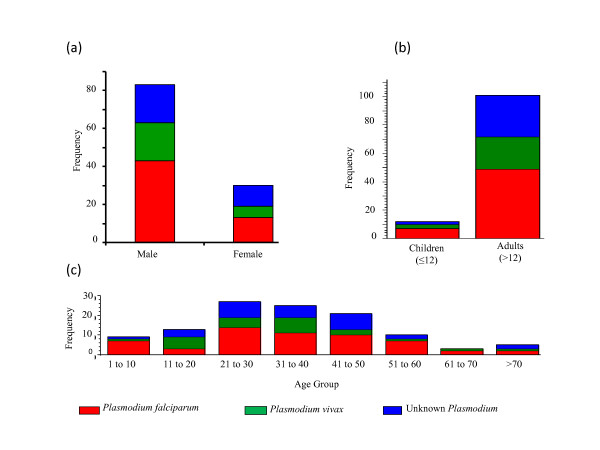
**Frequency of Plasmodium species by (a) gender, (b) children and adults, and (c) age distribution by Plasmodium species**.

A total of 113 patients diagnosed with cases of malaria were admitted to the hospital between July 2006 and July 2008. Figure 1a-c summarizes their demographic characteristics. Figure 1a shows the frequency distribution of patients by *Plasmodium *species and gender. The data reveal that the proportion of infections caused by *P. falciparum *is greater than *P. vivax *or unidentified *Plasmodium *for all age groups and genders. Most of the patients were males: 73% were male (n = 83) and 27% were female (n = 30). Figure 1b reveals that most of the patients were adults. There were only 12 paediatric patients (three girls and nine boys) and only two of the children were less than five years old. The results presented for this hospital in Delhi are consistent with the findings of other researchers [8,9]. Figure 1c indicates that the mean age of those malaria patients was 33.4 years with 46.0% (n = 52) of them being between 21 through 40 years old. The youngest patient was one year old while the oldest was 76 years old. For males, the mean age was 33.1 years with a maximum age of 76 and a minimum of one. Among females, the mean age was 34.5 years with a maximum age of 75 years and a minimum of one. A cautionary note, however, is warranted in generalizing from these data because available national-level data provide very limited information on age-specific prevalence for India [3]. This has the potential to introduce selection biased into this study.

Cases of malaria involving serious complications were extremely rare among our group of paediatric and adult patients. Complications were seen in just five cases of *P. falciparum*. All of them were male patients. Three of those patients (2.6%) developed cerebral malaria, and two cases had other complications. Out of the three cases with a diagnosis of cerebral malaria, two were children aged 1 and 8 years, respectively. The third case was a 72-year old male. Because only three cases (2.7%) were classified as very severe during the study period, it is not possible to provide further statistical analysis of severe malaria.

**Figure 2:**
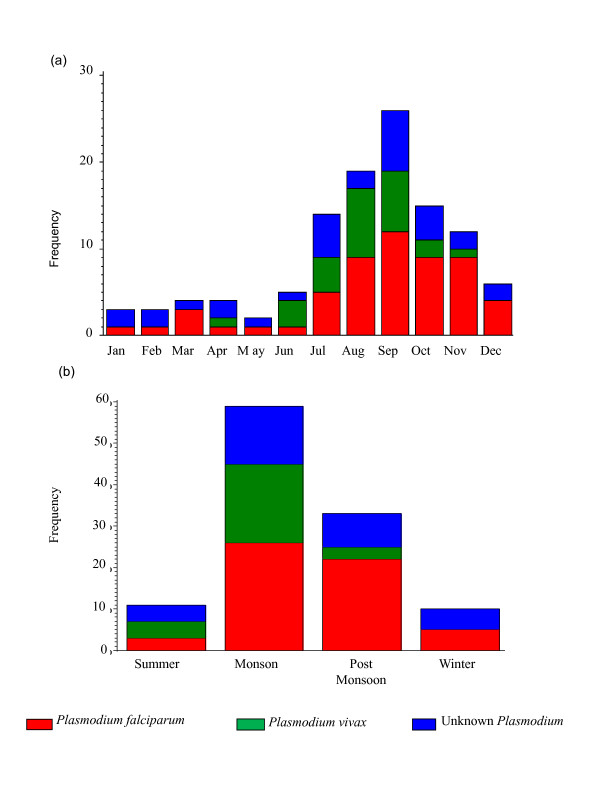
**(a) Monthly and (b) seasonal distributions of malaria cases by plasmodium species for patients in a private tertiary care hospital in Delhi**.

Figure 2 shows that malaria occurs throughout the year in India (Figure 2a). *Plasmodium falciparum *was the identified as the causative agent in 49.6% of the cases (n = 56) and *P. vivax *was the causative agent in 23.0% cases (n = 26). Finding *P. vivax *in a large percentage of the in-patient cases in a private, comprehensive-care hospital setting is notable because those cases were diagnosed using a combination of antigen tests and microscopy. Hence, there is confidence that the *Plasmodium *species are identified correctly. Malarial species was unidentified in 27.4% of the cases (n = 31). Monthly data reveals that *P. falciparum *occurs throughout the year. *Plasmodium falciparum *cases start increasing in July, peak in September, and then there is a gradual decline. *Plasmodium vivax *does not appear until June, peaks in August-September, and then shows a sharp decline in the October-November timeframe. With the exception of a single isolated case in April, no cases with *P. vivax *were recorded from December until June during the 24 months encompassed by this study.

Figure 2b illustrates the seasonal trends in malaria by *Plasmodium *species. During the monsoon season, falciparum cases constitute 44.1% of the patient load (n = 26) and vivax cases 32.2% of the total number of cases in this season (n = 19). In the post-monsoon, falciparum cases are predominant constituting 66.5% of the total malaria cases during this season (n = 22). *Plasmodium falciparum *was present in five of the ten cases observed during the winter months. No *P. vivax *infection was seen during the winter months. The summer season showed few cases of either *P falciparum *or *P. vivax*. Eleven cases were diagnosed during summer with *P falciparum *as the agent (27.3%; n = 30), while *P. vivax *was present in 36.4% of those cases (n = 4). The proportion of *P. vivax *cases compared to *P. falciparum *was highest during summer, with the largest number of cases occurring in June. The seasonal trend was consistent with the findings of [10].

Table 2 provides the seasonal distribution of malaria cases by species and provides the results of the χ^2 ^test for independence performed using Freeman-Halton exact test. Significant associations exist between Plasmodium species and seasons (p < 0.05). The proportions of malaria cases caused by each Plasmodium species vary by season. Malaria patients admitted during the monsoon and post monsoon seasons primarily have P. falciparum parasites. *Plasmodium falciparum *accounts for nearly half of all cases admitted during the study period. For this group of patients, *P. vivax *cases are most frequent in the monsoon season and decline notably in the post monsoon season. There appears to be no clear trend for malaria cases caused by unknown *Plasmodium*.

**Table 2:** Distribution of Plasmodium species by season, results for Freeman-Halton exact test for association between species and season, and test for trend by species (count, [percentage within a season], (percentage within a year))

**Species**	**Summer**	**Monsoon**	**Post Monsoon**	**Winter**	**Total**
*P. falciparum*	3 [27.27]	26 [44.02]	22 [66.67]	5 [50.00]	56 na
	(2.65)	(23.01)	(19.47)	(4.42)	(49.56)
*P. vivax*	4 [36.36]	19 [32.20]	3 [9.09]	0 [0.00]	26 na
	(3.54)	(16.81)	(2.65)	(0.00)	(23.01)
*P*. unidentified	4 [36.36]	14 [23.73]	8 [24.24]	5 [50.00]	31 na
	(3.54)	(12.39)	(7.08)	(4.42)	(27.43)
Total	11 na	59 na	33 Na	10 na	113 na
	(9.73)	(52.21)	(29.2)	(8.84)	(100)

Freeman-Halton exact test				p = 0.02	

Cochran-Armitage Z for *P. falciparum*			1.98	p = 0.03	
Cochran-Armitage Z for *P. vivax*			-3.06	p = 0.03	
Cochran-Armitage Z for *P*. unidentified			0.67	p = 0.29	

Note: totals not applicable marked by na

Table 2 also presents the proportion of cases caused by each *Plasmodium *species by season and list the result for temporal trends determined by the Cochran-Armitage test for trends. A statistically significant positive trend (p < 0.05) exists for *P. falciparum*. Proportions of malaria cases caused by this *Plasmodium *tend to increase from summer though post monsoon season, then decrease during the winter. A statistically significant negative trend (p ≤ 0.001) also exists for *P. vivax*, but the seasonal trend is the reverse of the one found for *P. falciparum*. The data indicate that malaria cases caused by *P. vivax *tend to decrease from summer through winter. No significant trend was found for the unknown *Plasmodium *species, which is what would be expected because the species is not identified.

A comparison between *P. falciparum *case detection for Delhi under the national surveillance programme (NVBDCP) and our data is provided in Table 3. The NVBDCP performs active and passive testing. Active testing occurs when health workers actively search for malaria cases house to house for malaria cases asking if household member have a fever and testing members who do. Passive sampling occurs when patients arrive at a primary health care facility with a fever and no obvious cause for the fever other than malaria. In both cases, blood slides are taken and examined using rapid diagnostic tests. According to the Operational Manual for Implementation of Malaria Programme 2009, positive blood slides from both active and passive sampling are discarded if positive and the patient is treated. If negative, the results may be confirmed by microscopy and microscopy is the preferred method of diagnosis if results can be obtained within 24 hours [11]. Note that positive blood slides result in treatment for falciparum malaria and this is the focus of the NVBDCP. The treatment for falciparum malaria also effectively treats malaria caused by other *Plasmodioum *species [11].

**Table 3:** Comparison of private hospital data with national data

**Private Hospital**			**National Data**		
**YEAR**	**No. of Malaria cases**	***Plasmodium falciparum***	**YEAR**	**No. of Malaria Cases**	***Plasmodium falciparum***
2006 July -December	60	31	2006	928	36
2007	35	15	2007	182	2
2008 Jan - July	18	10	2008	248	0

The national surveillance data indicate that the indo-gangetic plains, northern hilly states, north-western India and southern state of Tamil Nadu have less than 10% of cases caused by *P. falciparum *[3]. Given its location, it is expected that less than 10% of the cases in New Delhi would be caused by *P. falciparum *infections. The results fail to support this assumption. In 2006, for example, the national data indicate there were 36 reported cases of *P. falciparum*, while the data from just one private hospital had 31 cases with confirmed cases within the six-month period from July to December. Similarly, national surveillance data (NVBDCP) for the year 2007 for the State of Delhi shows that 668,761 blood slides were examined for malaria with 182 testing positive for malaria. Out of these, only two cases tested positive for *P. falciparum*, whereas these data show that in the same year, *P. falciparum *cases numbered 15. And, for all of 2008, the NVBDCP data shows no cases of *P. falciparum*, whereas the data presented from only one tertiary care hospital show 10 cases of *P. falciparum *malaria for the January to July 2008 timeframe. The difference in the proportion of falciparum cases between the NVBDCP and the private hospital is a cause for serious concern with respect to the obvious under-reporting in the national data.

The increased proportion of *P. falciparum *malaria in this single, private hospital compared to the NVBDCP may be attributed to the disease's severity, which required admission or referral. Prior studies have shown that tertiary care hospitals treat more *P. falciparum *malaria cases [12-14]. Another potential reason for the difference may be the reliance of the private hospital on more stringent laboratory diagnostic tests. The data indicate that the *P. falciparum *variant was the causative agent in almost 50% of the cases, while *P. vivax *was identified as the causative agent in slightly less than 25% of the cases with remainder unknown. Because positive species delineation was done using microscopy to corroborate antigen tests in all cases, this may partially account for why the *P. falciparum *burden is underestimated in the NVBDCP surveillance data for India which relies primarily on blood slide examination as a diagnostic tool.

## Conclusion

Probability sampling was not used to assess the underlying malaria burden in the NCR region or the private health care facility within the region. Instead, the cases were essentially self-selected based on the initial clinical diagnosis by physicians or health practitioners. Therefore, one needs to be cautious in making overly broad generalizations due to inherent sampling bias.

The analysis of patterns in falciparum and vivax malaria among patients in a private comprehensive-care, multi-specialty hospital in New Delhi reveals that *P. faciparum *was the dominant cause of cases requiring treatment in the facility on an overall basis. *Plasmodium vivax *also caused a large proportion of the cases presenting clinically in the summer and monsoon seasons. The proportion of *P. falciparum *malaria cases tends to be greatest during the post-monsoon season, while the proportion of *P. vivax *malaria cases tends to be greatest in the monsoon season. This seasonal variation between the *Plasmodium *species causing malaria is worth noting.

As noted above, the analysis presented is constrained by the data-selection process. In all cases (private and NVBDCP), only individuals know to have malaria or with a fever and suspected to have malaria were tested for malaria. This limits the generalizability of the results. Interestingly enough, despite this caveat, under-reporting of *P. falciparum *malaria cases was evident when the data from the private hospital were compared with data from the national surveillance programme for approximately same period for the NCR region. While the data indicate a large number of *P. falciparum *malaria cases were treated at this single hospital, the national data reported a smaller number of malaria cases caused by *P. falciparum *for the region. This is a startling discrepancy and demonstrates clearly the importance of routinely collecting data from private health care facilities in order to delineate more fully patterns of malaria in India. Moreover, unless such data from private health care facilities are systematically integrated into surveillance programmes, the size of the malaria problem requiring health care services may be misestimated.

## Competing interests

The authors declare that they have no competing interests.

## Authors' contributions

SG conceived the study, obtained the data from India, performed statistical analysis, and drafted the article. JTG assisted with statistical analysis and manuscript authoring. RJN assisted with manuscript authoring. JLR directed and designed the study, and assisted with manuscript authoring. All authors read and approved the final manuscript.
